# Association of rs3787016 in Long Non-coding RNAs *POLR2E* and rs2910164 in MiRNA-146a with Prostate Cancer: A Systematic Review and Meta-analysis

**Published:** 2018-05

**Authors:** Shuaishuai HUANG, Hui CUI, Zhongguan LOU, Xue WANG, Liangliang CHEN, Zhenhua XIE, Michael HEHIR, Xuping YAO, Yu REN, Dong CEN, Guobin WENG

**Affiliations:** 1. Laboratory of Kidney Carcinoma, Urology and Nephrology Institute of Ningbo University, Ningbo Urology and Nephrology Hospital, Ningbo, Zhejiang 315000, China; 2. Dept. of Medical School, Ningbo University, Ningbo, Zhejiang 315211, China

**Keywords:** SNP, rs3787016, rs2910164, Prostate cancer, Meta-analysis, lncRNA POLR2E, miRNA-146a

## Abstract

**Background:**

The aim of this study was to conduct a meta-analysis to estimate the association between the two SNPs and PCa risk.

**Methods:**

Medline, Embase, Scopus, PubMed, Web of Science, Wan Fang Database and Chinese Zhi Wang Database were searched for the association of the two SNPs with susceptibility to PCa. The effect size was pooled by odds ratios (ORs) and 95% confidence intervals (95% CIs).

**Results:**

Nine case-control studies, 5 on rs3787016 and 4 on rs2910164, were included. As regards rs3787016, an increased risk of PCa was identified in all genotype models (T versus C: OR = 1.18, 95% CI 1.11–1.25; CT versus CC: OR = 1.17, 95% CI 1.08–1.26; TT versus CC: OR = 1.41, 95% CI 1.22–1.63; TT + CT versus CC: OR = 1.20, 95% CI 1.12–1.30; TT versus CT + CC: OR = 1.32, 95% CI 1.15–1.52). However, no significant association was found between rs2910164 and PCa risk in any genetic models, in fact a trend of reduced risk could be seen (C versus G: OR = 0.91, 95% CI 0.79–1.05; GC versus GG: OR = 0.93, 95% CI 0.74–1.18; CC versus GG: OR = 0.70, 95% CI 0.47–1.02; CC + GC versus GG: OR = 0.90, 95% CI 0.73–1.12; CC versus GC + GG: OR = 0.78, 95% CI 0.56–1.08). Besides, in analysis of subgroups by source of controls, the decreased results were observed in studies using population-based controls.

**Conclusion:**

lncRNA *POLR2E* polymorphism rs3787016 is associated with a significantly increased risk of PCa, while a trend of reduced risk appears with mir-146a polymorphism rs2910164.

## Introduction

Prostate cancer (PCa) is the most common non-skin malignancy in male populations, ranking second for cancer-related deaths in the US and EU male populations (1, 2). In the clinical field, the early stage of developing PCa is common without any symptoms while the diagnosed cases are often identified in advanced stages with poor outcomes. With surgical and medical advances, early stages PCa is often cured by surgical resection, local radiation or ablation therapies, while for advanced stage disease, chemotherapy and hormone therapy have made progress. However, due to metastases to other parts of the body, particularly the bones and lymph nodes, the survival rate of advanced PCa remains seriously decreased (3). Thus, it is critical to identify the susceptible population and potential external factors to prevent and control the development of PCa. Evidence exists that ethnicity, age, and heredity can contribute to the progress of PCa (4). Besides, progression of PCa might be associated with altered expression of multiple oncogenes and tumor suppressors. In regulating these genes, microRNAs or lncRNAs play important roles (5, 6). Besides, eligible studies have revealed single nucleotide polymorphisms related to the PCa tumorgenesis (7, 8).

The SNP rs3787016 locates at an lnc RNA spanning 364kb at 19p13, also localizes to an intron of *POLR2E* gene, encoding a subunit of RNA polymerase II and responses to synthesizing messenger RNA. The association between rs3787016 variants and PCa susceptibility was identified, however, the results were inconsistent (1, 9, 10). Another SNP, the has-miR-146a polymorphism rs2910164, located in the middle of the stem hairpin in the microRNA, leads to a G: U pair to C: U mismatch in the stem structure, possibly affecting the amount of mature miR-146a and eventually predisposition to PCa (11). rs2910164 C variant played a protective effect on PCa risk in a southern Chinese Han population (11), whereas this protective effect was not seen in Serbian (12), North Indian (13) and Iranian populations (14). These SNPs (rs3787016 and rs2910164) display discrepant results. Therefore, it is essential to make it clear whether these two SNPs are associated with PCa.

In this study, we aimed to investigate the susceptibility of the two polymorphisms with PCa globally, to provide a more comprehensive estimation of associated risk. Herein, we conducted a meta-analysis to clarify the impact of the two polymorphisms on the risk of developing PCa.

## Methods

### Search strategy

Medline, Embase, Scopus, PubMed, Web of Science, Wan Fang Database and Chinese Zhi Wang Database were searched using the following terms: (“Prostate cancer” OR ”PCa” OR “Prostate carcinoma”) AND (“rs3787016” OR “rs2910164”) by two independent investigators (Wang and Chen). Various combinations of the terms were also used. Literature search data were last updated on May 10, 2016. No limitations were considered.

### Criterion for study selection and Data Extraction and Synthesis

The selected studies met the following criteria: (1 studies that performed the associations of lncRNA *POLR2E*/has-miR-146a and PCa; (2 detailed genotype data in case-control, cohort groups and GWAS could calculate or provide the odds ratios (ORs) with 95% confidence intervals (CIs). Exclusion criteria: (1 no detailed genotype data; (2 comment, review, meta-analysis, and editorial; (3 for multiple publications from the same population/area, only the largest sample was included.

However, the data of eligible studies were extracted independently, including the following contents: name of first author, year of publication, the characteristics of cases and controls, country of origin, number of cases and controls for lncRNA *POLR2E*/has-miR-146a genotypes, respectively (some data were calculated according to the minor allele frequency and sample size). Different ethnicity was classified as Caucasian and Asian, and source of controls as population-based, hospital-based and unknown. Extracted data were checked and consensus reached by two authors, if dissent existed, the authors rechecked the original data and held a discussion to reach a consensus. If the dissent still existed, a third investigator assisted by adjudicating the disagreements.

### Statistical Analysis

We conducted our meta-analysis according to the PRISMA checklists and followed the guidelines (15). To evaluate the strength of the association between rs3787016/rs2910164 polymorphism and PCa risk, the pooled odds ratios (ORs) with 95% confidence intervals (CIs) were calculated. Pooled ORs were performed for allelic comparison (lncRNA *POLR2E*: T versus C, miR-146a: C versus G), heterozygote model (lncRNA *POLR2E*: CT versus CC, miR-146a: GC versus GG), homozygote model (lncRNA *POLR2E*: TT versus CC, miR-146a: CC versus GG), dominant model (lncRNA *POLR2E*: CT + TT versus CC, miR-146a: CC + GC versus GG), recessive model (lncRNA *POLR2E*: TT versus CC + CT, miR-146a: CC versus GC + GG), respectively.

We used *Z*-test to determine the pooled OR and 95% CI, and *P*<0.05 was regarded as statistically significant. Statistical heterogeneity among studies was assessed by the *Q* statistic (significance level of *P*<0.1) and *I^2^* statistic (greater than 50% as evidence of significant inconsistency) (16). The *Q* test and *I^2^* statistic were applied to test variation according to heterogeneity. When *P* value of heterogeneity (*P_H_*) was no more than 0.1 (*P_H_* ≤ 0.1), random effects model was used, and when *P_H_* was > 0.1, fixed effects model was used (17). Sensitivity analysis was also tested by removing one study at a time, to evaluate the effect of removal and effect of size of each study on the homogeneity of the whole. In addition, subgroup analyses were stratified by ethnicity and source of controls. Publication bias was investigated with Begg’s funnel plots and further assessed by the Egger’s regression test (18, 19). When an asymmetric plot was shown or Egger’s test was *P*<0.05, we considered it as a significant publication bias. Besides, Hardy–Weinberg equilibrium (HWE) was implemented to identify the effective records in our study (20). All analyses were performed with Stata 12.0 software (StataCorp, CollegeStation, TX, USA). A two-tailed *P*<0.05 was regarded as significant, except for specified conditions, which a certain *P*-value was declared.

## Results

### Search results and quality assessment

**Fig. 1:**
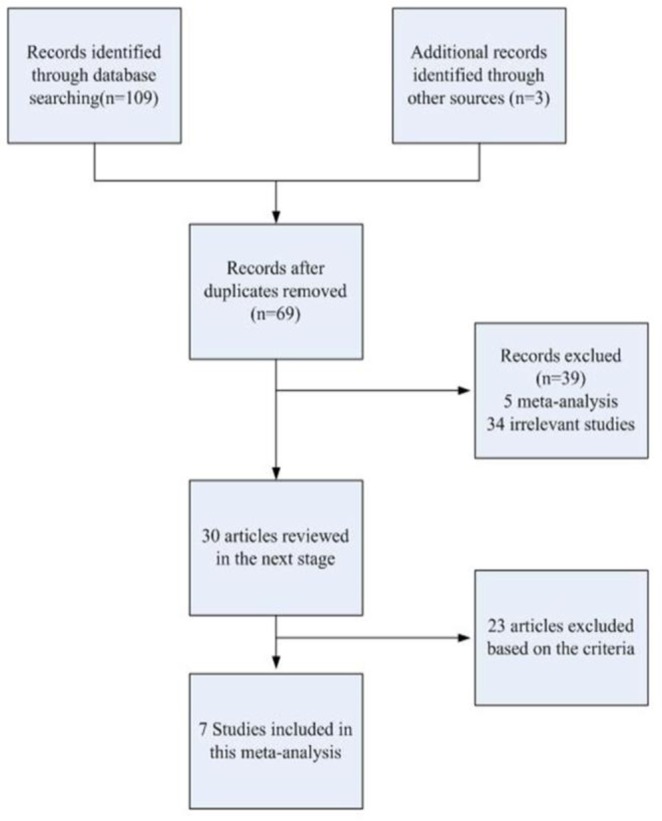
Process of study selection

According to inclusion and exclusion criteria, 7 articles were identified (Fig. 1). One multi-center (9), recruited from 3 different regions, were included as 3 separate studies. Therefore, 5 case-controls (5472 cases and 6145 controls) and 4 case-control (865 cases and 892 controls) respectively were collected to assess the association of rs3787016 and rs2910164 with PCa risk (1, 9, 10, 12–14, 21) (Table 1).

**Table 1:** Characteristics of the studies related to the effects of rs3787016/rs2910164 and PCa

***Study ID***	***Year***	***Country***	***Ethnicity***	***Source of controls***	***case***			***Control***			**P *for HWE***
**Long noncoding RNA *POLR2E* rs3787016 C > T**					CC	CT	TT	CC	CT	TT	
JHH study	2009	USA	Caucasian	Unknown	1016	751	139	1805	1109	170	0.9839
CGEMS study	2007	USA	Caucasian	Unknown	635	458	83	634	403	64	0.9969
Replication study	2011	USA	Caucasian	Unknown	607	431	76	492	288	42	0.9861
Cao DL study	2004	China	Asian	PB	313	513	189	55	44	7	0.064
Nikolić ZZ study	2013	Serbia	Caucasian	PB	140	100	21	105	194	71	0.6481
**miRNA-146a rs2910164 G>C**					GG	GC	CC	GG	GC	CC	
Bin Xu study	2010	China	Asian	PB	68	135	48	54	150	76	0.1913
George GP study	2011	India	Asian	PB	76	69	4	116	107	7	0.0024
Nikolic ZZ study	2014	China	Caucasian	PB	184	90	12	129	63	7	0.8385
Hashemi M study	2016	Iran	Caucasian	PB	25	131	13	24	147	21	0.000

PB: population based; Unknown: not mentioned in the original reference

### Association between rs3787016 and risk of PCa

**Fig. 2:**
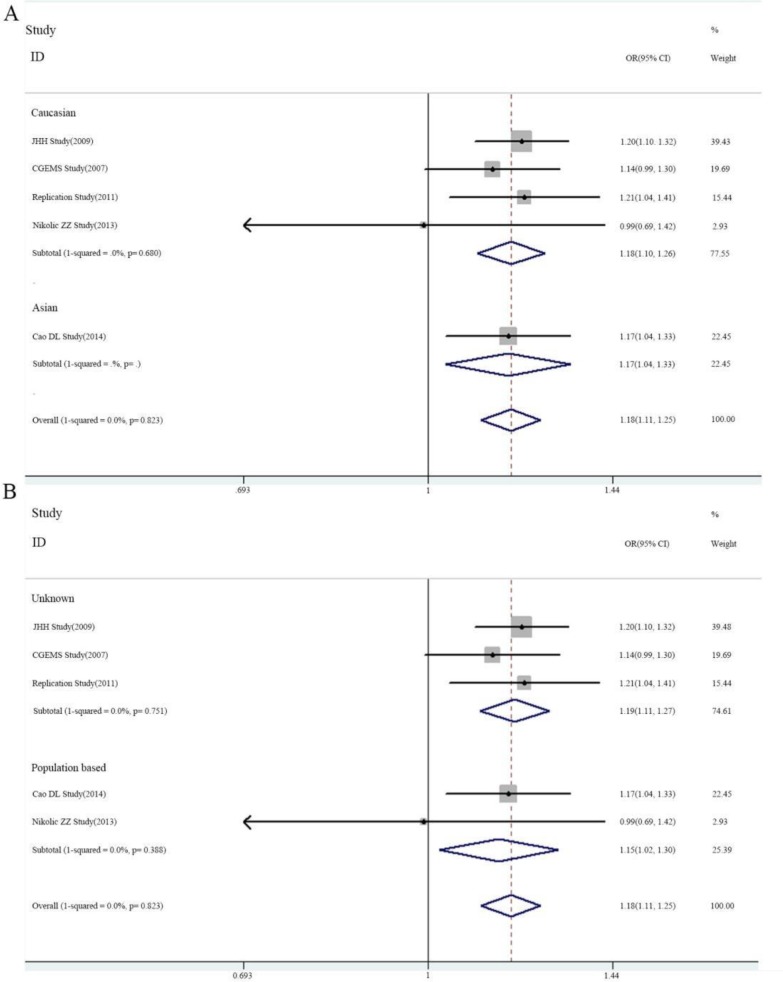
Forest plots of association between rs3787016 polymorphism and PCa stratified by ethnicity (A) and source of controls (B)

We analyzed the association between lncRNA *POLR2E* rs3787016 polymorphism and PCa risk. No significant heterogeneity was identified by *Q-*test and *I^2^* statistic in any genetic models. Then, the fixed-effects model was used. Overall, an increased risk was identified in genotypes carrying the T allele (T versus C: OR = 1.18, 95% CI 1.11–1.25, *P_H_* = 0.823; CT versus CC: OR = 1.17, 95% CI 1.08–1.26, *P_H_* = 0.747; TT versus CC: OR = 1.41, 95% CI 1.22–1.63, *P_H_* = 0.974; TT + CT versus CC: OR = 1.20, 95% CI 1.12–1.30, *P_H_* = 0.770; TT versus CT + CC: OR = 1.32, 95% CI 1.15–1.52, *P_H_* = 0.993) (Table 2 and Fig. 2). Besides, subgroup analysis was performed to examine the effects of ethnicity and source of controls (Table 2).

**Table 2:** Subgroup analysis of the association between rs3787016 and PCa

	***N***	***OR***	**P_H_**	***OR***	**P_H_**	***OR***	**P_H_**	***OR***	**P_H_**	***OR***	**P_H_**
		T/C		CT/CC		TT/CC		TT+CT/CC		TT/CT+CC	
**Overall**	5	1.18 (1.11–1.25)	0.823	1.17 (1.08–1.26)	0.747	1.41 (1.22–1.63)	0.974	1.20 (1.12–1.30)	0.770	1.32 (1.15–1.52)	0.993
**Sources of controls**											
Unknown	3	1.19 (1.11–1.27)	0.751	1.19 (1.09–1.30)	0.836	1.41 (1.19–1.68)	0.846	1.22 (1.12–1.32)	0.781	1.32 (1.11–1.57)	0.895
PB	2	1.15 (1.02–1.30)	0.388	1.08 (0.90–1.29)	0.391	1.41 (1.09–1.81)	0.692	1.15 (0.97–1.36)	0.333	1.33 (1.06–1.65)	0.871
**Ethnicity**											
Caucasian	4	1.18 (1.10–1.26)	0.680	1.18 (1.08–1.28)	0.635	1.40 (1.18–1.67)	0.923	1.21 (1.11–1.31)	0.617	1.32 (1.11–1.56)	0.971
Asian	1	1.17 (1.04–1.33)	—	1.12 (0.92–1.36)	—	1.43 (1.10–1.86)	—	1.19 (0.99–1.43)	—	1.33 (1.06–1.69)	—

PB: population based; HB: hospital based

### Association between rs2910164 and risk of PCa

**Fig. 3:**
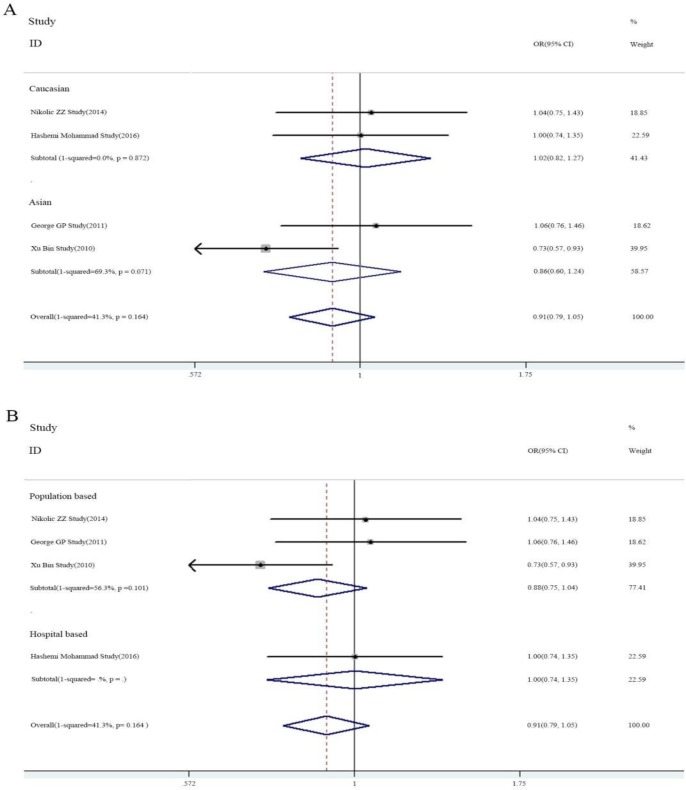
Forest plots of association between rs2910164 polymorphism and PCa stratified by ethnicity (A) and source of controls (B)

Four included studies were examined to determine the association of rs2910164 and PCa. Fixed-effects model was used recognizing no significant heterogeneity. The meta-analysis showed no significant association in any genetic model between rs2910164 and PCa risk, but a trend of reduced risk could been seen (C versus G: OR = 0.91, 95% CI 0.79–1.05, *P_H_* = 0.164; GC versus GG: OR = 0.93, 95% CI 0.74–1.18, *P_H_* = 0.472; CC versus GG: OR = 0.70, 95% CI 0.47–1.02, *P_H_* = 0.270; CC + GC versus GG: OR = 0.90, 95% CI 0.73–1.12, *P_H_* = 0.250; CC versus GC + GG: OR = 0.78, 95% CI 0.56–1.08, *P_H_* = 0.362) (Fig. 3).

In subgroup analysis examining ethnicity and sources of controls, a trend of decreased risk was observed in Asians and in studies using population-based controls. Moreover, after excluding the two studies (13, 14), not meeting the HWE, the reduced risk was still shown (Table 3).

**Table 3:** Subgroup analysis of the association between rs2910164 and PCa

	***N***	***OR***	**P_H_**	***OR***	**P_H_**	***OR***	**P_H_**	***OR***	**P_H_**	***OR***	**P_H_**
		C/G		GC/GG		CC/GG		CC+GG/GG		CC/GC+GG	
**Overall**	4	0.91 (0.79–1.05)	0.164	0.93 (0.74–1.18)	0.472	0.70 (0.47–1.02)	0.270	0.90 (0.73–1.12)	0.250	0.78 (0.56–1.08)	0.362
**Sources of controls**											
PB	3	0.88 (0.75–1.04)	0.101	0.094 (0.74–1.19)	0.295	0.64 (0.42–0.97)	0.250	0.91 (0.72–1.14)	0.129	0.71 (0.50–1.02)	0.468
HB	1	1.00 (0.74–1.35)	—	0.86 (0.47–1.57)	—	1.13 (0.43–3.02)	—	0.87 (0.48–1.60)	—	1.30 (0.56–2.98)	—
**Ethnicity**											
Caucasian	2	1.02 (0.82–1.27)	0.872	0.96 (0.69–1.13)	0.669	1.17 (0.59–2.32)	0.934	0.98 (0.71–1.35)	0.670	1.25 (0.67–2.34)	0.907
Asian	2	0.86 (0.60–1.24)	0.071	0.91 (0.67–1.22)	0.131	0.54 (0.34–0.87)	0.425	0.85 (0.50–1.45)	0.062	0.65 (0.44–0.98)	0.699
**Exclude the study not meeting HWE**											
	2	0.85 (0.61–1.21)	0.085	0.86 (0.64–1.44)	0.254	0.61 (0.39–0.95)	0.114	0.824 (0.63–1.09)	0.103	0.70 (0.48–1.02)	0.227

PB: population based; HB: hospital-based

### Sensitivity analysis

**Fig. 4:**
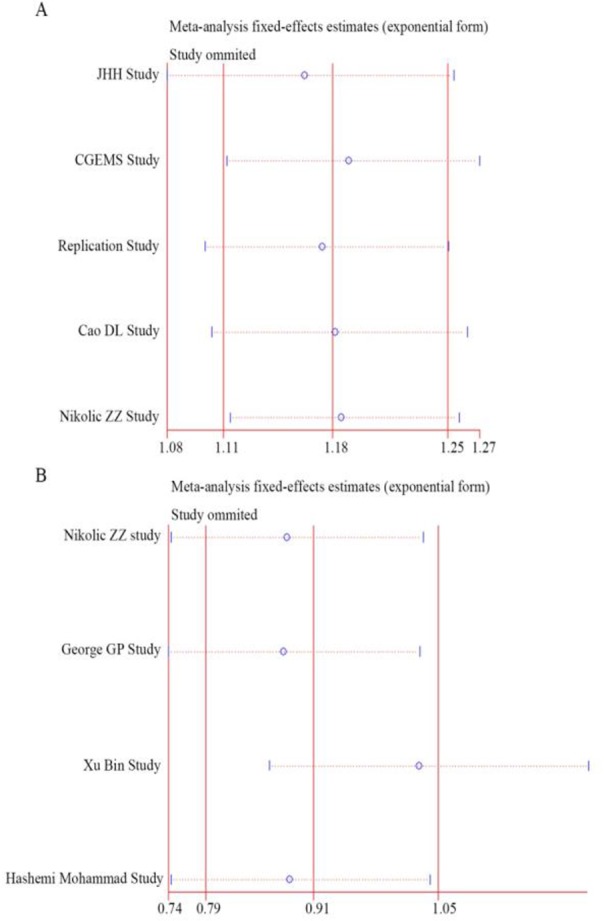
Sensitivity analysis of rs3787016 (A) and rs2910164 (B)

We performed a sensitivity analysis to examine the stability of the pooled ORs with the effect of the individual studies. With removal of individual study results from the analysis for rs3787016 and rs2910164, the pooled ORs remained significantly consistent (Fig. 4A, B).

### Publication bias

**Fig. 5:**
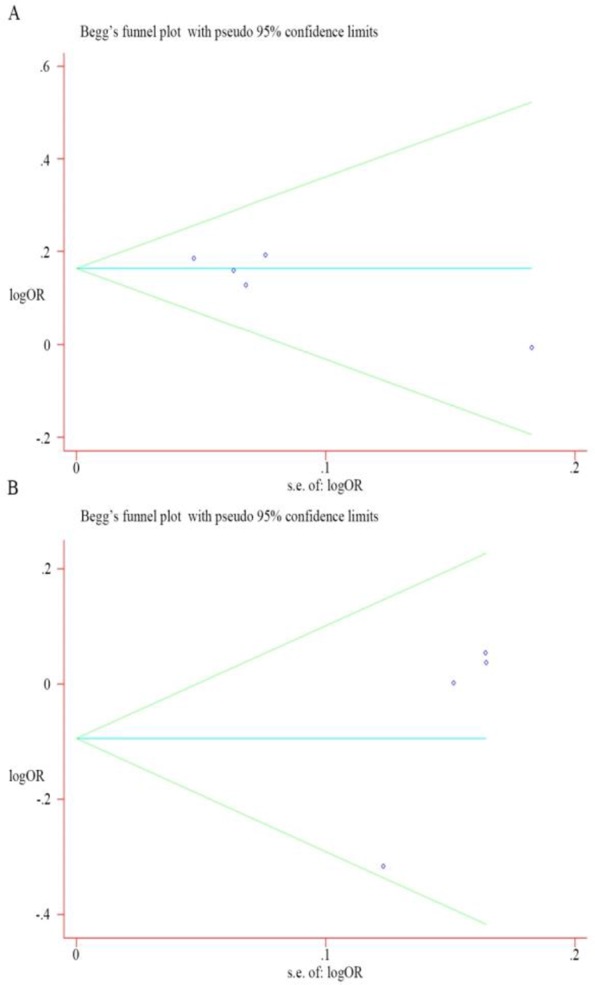
Begg’s funnel plot of publication bias test for rs3787016 (A) and rs2910164 (B)

We used Begg’s test and Egger’s test to assess publication bias. No evidence of publication bias were shown in all genotype models of rs3787016 (*P*-value not is shown), and all genotype models of 2910164 except for the allele model (Begg’s funnel plot: *P*=0.174, C versus G; Egger’s regression test: *P*=0.016, C versus G). (Fig. 5).

## Discussion

In this meta-analysis, we investigated 5 eligible case-control studies including 5472 cases and 6145 controls for rs3787016, and 4 studies including 865 cases and 892 controls for rs2910164. The results showed an increased risk for PCa in genotypes carrying the T allele in rs3787016, however, a potential trend of reduced risk between rs2910164 and PCa was observed. Non-coding RNA includes highly abundant and functionally important RNAs, such as microRNAs and long non-coding RNAs. Alterations of microRNAs or non-coding RNAs contribute significantly to the tumorigenesis of prostate cancer (22, 23). SNPs in microRNAs and non-coding RNAs regions may affect their functions through altering their maturation and expression as oncogenes. LncRNAs can serve as the precursors of small non-coding RNAs to produce microRNAs and endogenous small interfering RNA or acts as a “microRNA sponge” to inhibit microRNA activity (24, 25). Several lncRNAs also play vital roles, activating or suppressing oncogenes (26, 27). The A allele of rs3787016 was associated with a 1.19-fold (95% confidence interval: 1.11–1.27) increased PCa risk, then they conducted a meta-analysis of two GWAS and a replication study, and eventually identified a novel PCa risk locus rs3787016 at 19p13 in Caucasians. Regretfully, no subgroup analysis was done to assess the role of the control source and ethnicity (9). After that, rs3787016 was associated with PCa in an eastern Chinese population (1). In contrast, a lack of association between rs3787016 and PCa risk was found in a Serbian population (10). Our meta-analysis further pooled the above studies and recognized a significant association between rs3787016 and PCa risk. As for source of controls, three studies (GWAS study) (9) did not mention the source of control, and in two studies (1, 10) controls were population-based. Given the limitation of studies as regards sources of controls, more studies in different populations should be carried out to identify reliable effects.

The SNP rs2910164 is shown to affect biogenesis of mature miR-146a and is located in the “seed” region of the passenger strand (21). However, there are controversial results in various cancers regarding the association between hasmiR-146a SNP (rs2910164) and mature miR-146a production (11, 28). It is also not clear whether homozygous GG or CC genotype is associated with a higher mature miR-146a expression. The roles of rs2910164 were researched in the development of cancers. Their results showed no observed association between rs2910164 and cancer risk in the overall group. Interestingly, in stratified analysis, they found that either the rs2910164 C allele or the CC genotype was protective against bladder cancer, prostate cancer, cervical cancer, and colorectal cancer, whereas it was a risk factor for papillary thyroid carcinoma and squamous cell carcinoma of the head and neck (SCCHN) (29). In our meta-analysis, we did not find a significant association between miR-146a (rs2910164) and PCa, but a trend towards reduced risk could be seen (Table 3). Moreover, a significantly reduced risk of PCa was shown in the homozygote model in population-based and Asian studies and studies meeting the HWE criteria, indicating that rs2910164 polymorphism might play an important role in PCa development, consistent with previous meta-analyses (29–31).

When we performed sensitivity analysis for studies on rs3787016, the outcomes remain unchanged by deleting each study once in each genetic model, indicating that no heterogeneity was found in the included studies. Begg’s funnel plot or Egger’s regression test was used to checking the publication bias, and no bias was found.

Significant heterogeneity was found, however, in studies with rs2910164 and PCa in three genetic models (two Asian studies and one not meeting HWE, *P_H_* < 0.10), so the random effect model was used in our meta-analysis. In addition, a publication bias existed between rs2910164 allele model and PCa risk by Egger’s regression test (C versus G: *P*=0.016). This may have been related to the limited number of studies and subjects in this category and the well-known bias towards reporting of positive studies (32, 33).

Some limitations can be identified in our meta-analysis. Firstly, the number of included studies was limited, which might influence the reliability of the findings. Secondly, two studies of miRNA-146a rs2910164 did not meet HWE criteria (13, 14), causing a restriction of further analyses. Still, our results are supported by existing literature, and further studies are urgently needed in global multi-centered populations.

## Conclusion

T allele of rs3787016 polymorphism might increase the susceptibility of PCa in both Asians and Caucasians. However, no significant association was found for miR-146a (rs2910164) and PCa, but a trend of reduced risk could be seen. Multi-center studies with larger sample size are required to assess these potential genetic risk factors for the development of PCa.

## Ethical considerations

Ethical issues (Including plagiarism, informed consent, misconduct, data fabrication and/or falsification, double publication and/or submission, redundancy, etc.) have been completely observed by the authors.
